# Association between amniotic fluid evaluation and fetal biometry: a prospective French “Flash” study

**DOI:** 10.1038/s41598-018-25497-3

**Published:** 2018-05-04

**Authors:** Florent Fuchs, Safa Aouinti, Manel Souaied, Valentin Keller, Marie-Christine Picot, Nicolas Fries, Jean-Marc Ayoubi, Olivier Picone

**Affiliations:** 1 Department of Obstetrics and Gynecology, Montpellier University Hospital Center, 371 Avenue du Doyen Gaston Giraud, Montpellier, France; 2 Inserm, CESP Centre for research in Epidemiology and Population Health, U1018, Reproduction and child development, Villejuif, France; 3 Clinical Research and Epidemiology Unit (URCE), CHU Montpellier, Univ Montpellier, Montpellier, France; 4 Department of Obstetrics and Gynecology, Hopital Foch, 40 rue Worth, Suresnes, France; 5 Collège Français d’Echographie Foetale, CFEF, France; 6 EA2493, UFR des sciences de la santé Simone Veil, Université Versailles Saint Quentin en Yvelines, Versailles, France; 7 Department of Obstetrics and Gynecology. Louis Mourier Hospital, Paris Nord Val de seine University Hospitals, APHP, Paris-Diderot University, 178 rue des Renouillers Colombes, Paris, France

## Abstract

We aimed to study the association between three different methods of assessing the amount of amniotic fluid (subjective method (SM), deepest vertical pocket (DVP) and amniotic fluid index (AFI)) and estimated fetal weight (EFW) (in percentile or Z-score) after adjustment on maternal-fetal parameters. We performed a nationwide cross-sectional study through the French network of obstetric sonographers using the “flash” study method and including low-risk singleton pregnancies from 18–40 weeks. Crude and adjusted odds ratio were computed after stratification upon 2^nd^ and 3^rd^ trimester of pregnancy. 1667 ultrasound scans performed by 65 operators were included. Only Z-score of EFW was significantly associated with SM in both trimesters. For DVP and AFI, Z-score of EFW and male fetal gender was significantly associated with them in 2^nd^ trimester. In the 3^rd^ trimester, both Z-score of EFW and large (LGA) or small for gestational age (SGA) fetus were significantly associated with AFI. and DVP. Overweight woman and class I obesity women were also significantly associated with DVP modification. In conclusion, all three methods of amniotic fluid evaluation are significantly associated to estimated fetal weight. DVP and AFI appeared equivalent except that maternal-fetal factors seemed to have a higher impact in DVP than AFI.

## Introduction

The evaluation of amniotic fluid during a routine ultrasound scan can be performed in three ways: the subjective way, the measurement of the single deepest vertical pocket, or the evaluation of amniotic fluid index. The subjective method consists of visually estimating the amniotic fluid pockets during an ultrasound scan. The accuracy of this assessment will rely strongly on the experience of the sonographer^1^. The deepest vertical pocket evaluation consists of measuring the largest pocket free of fetal structures or cord with the ultrasound probe positioned parallel to sagittal plane^2,3^. The amniotic fluid index is the sum of the four largest vertical pockets of amniotic fluid, measured in each of the 4 uterus quadrants defined relative to the umbilicus^4,5^. Several studies have reported that the amniotic fluid index was dependent on gestational age^6,7^. Even though none of these methods is really satisfactory, discovering an abnormal amount of amniotic fluid during pregnancy may change the perinatal care. In fact, finding a decrease amount of amniotic fluid during pregnancy might be due to either a pathological situation such as preterm premature rupture of membranes or a malformation/dysfunction of the fetal urination, or might also be physiological, but needing ultrasound survey of the evolution.

Intuitively, many sonographers interpret the amount of amniotic fluid based on the estimation of fetal weight performed at the same time. It seems reassuring to have a more abundant amniotic fluid when the fetus has a greater weight. Indeed, fetal renal perfusion increases when increasing fetal weight^8^. Therefore, we can assume that there might be a correlation or an association between fetal weight estimation and the quantity of amniotic fluid. Such an association could indicate that the amniotic fluid assessment should consider the fetal parameters to make it more precise. A review of the existing literature showed conflicting results on the possible correlation between the amount of amniotic fluid and fetal weight estimation^9–12^. However, these studies were all unicentric, based only on a small number of patients (from 90 to 400 patients), mainly retrospective and did not analyze the influence of other factors. Ultrasound scans were only performed during the 3^rd^ trimester of pregnancy and did not assess the single deepest vertical pocket method even though it has been demonstrated that this method was more reliable than the amniotic fluid index that would result in an increase in the false positive rate for a diagnosis of oligohydramnios^10,13,14^. Besides fetal parameters, maternal characteristics such as maternal age or body mass index may also influence the amount of amniotic fluid.

Given the aforementioned considerations, the objective of our study is to determine if there is an association between the different methods of assessing the amniotic fluid and the estimated fetal weight in crude and after adjustment on maternal or fetal characteristics.

## Material and Methods

### Study design

This study is a national prospective, multicentric, observational study, performed under the methodology of “Flash” studies^15–17^. Flash studies are pragmatic, short and very focused studies conducted without modifying the routine clinical practice. They are conducted over the countrywide network of sonographers who are members of the French College of Fetal Ultrasound (CFEF - College Français d’Echographie Foetale). We invited sonographers first to take an online training course (www.cfef.org) reviewing the aims of the study, the inclusion criteria and the methodology. Only sonographers who completed the course and passed the final test were eligible to participate in the study. From February 2^nd^ 2016 to February 15^th^ 2016, sonographers were asked to assess during their routine ultrasound scan the amount of amniotic fluid according to the three methods (subjective/single deepest vertical pocket/amniotic fluid index). After receiving patient approval, the sonographer accessed, through his/her personal login codes, to a patient’s individual electronic file report on the CFEF website, dedicated to the study, and with which he/she fulfilled patient data. Each pregnant woman contributed to the study with a single ultrasound scan and was prospectively and consecutively included over time. Pregnancy dating was based on the crown-rump length measurement in the first trimester, as recommended by the French College of Obstetrics and Gynecology (CNGOF)^18,19^.

### Population

Inclusion criteria were: women over 18 years, carrying a singleton pregnancy without congenital malformation at a gestational age from 18 weeks to 40 weeks of gestation, with an uncomplicated pregnancy and who consent to participate in the study. Non-inclusion criteria were the existence of a pregnancy complication listed below: hypertensive disorders of pregnancy (which includes gestational hypertension, preeclampsia, severe preeclampsia, and eclampsia), gestational diabetes, stillbirth, cholestasis and threatened preterm labor. Gestational hypertension was defined by a systolic pressure of 140 mm Hg or higher or a diastolic pressure of 90 mm Hg or higher on two separate occasions after 20 weeks of gestation in the absence of proteinuria. Preeclampsia was defined as gestational hypertension with either proteinuria 300 mg or more in a 24-hour sample or, if a 24-hour sample was not available, 2+ or higher on dipstick testing, or a urinary protein-to-creatinine ratio of 0.03 g/mmol or more.^20–22^ Severe preeclampsia was defined as preeclampsia associated with any adverse criteria: systolic pressure of 160 mm Hg or higher or a diastolic pressure of 110 mm Hg, or renal impairment (oliguria <500 mL/24 hours, or creatinine >135 micromol/L, or proteinuria >3 g/24 hours), or pulmonary edema, or persistent epigastric bar pain, or HELLP syndrome, or persistant neurological signs or abruptio placentae^22^. Eclampsia was defined by a convulsive tonic-clonic seizure in a context of hypertensive pathology of pregnancy^22^. Gestational diabetes was defined by one abnormal value on the 75 g oral glucose tolerance test according to thresholds from the HAPO study (fasting ≥ 5.1 mmol/l, 1 hour ≥ 10.0 mmol/l, 2 hours ≥ 8.5 mmol/l)^23^. Cholestasis was defined as symptoms of pruritus that typically include the palms and soles, as well as elevated bile acid levels^24^. Threatened preterm labor was defined as regular uterine contraction associated with cervical length shortening <25 mm on transvaginal scan^25^.

The sonographer should collect the following information: maternal age, parity, gravidity, Body mass Index (BMI) before pregnancy (defined as weight in kg/(height in meter)^2^), smoking status, gestational age at scan, fetal gender, bi-parietal diameter (BPD), fetal head circumference (HC), abdominal circumference (AC), femur length (FL), estimated fetal weight (EFW) and quantity of amniotic fluid according to the three methods. Fetal measurements should follow quality criteria previously described^26,27^. Estimated fetal weight (EFW) was obtained with the 4 parameters Hadlock formula^28^ and EFW percentile was derived by using Hadlock reference charts^29^. Regarding amniotic fluid evaluation, the sonographer began with the subjective method (SM) consisting in visually estimate the amniotic fluid pockets during the ultrasound scan. We asked the sonographer to report and classify the findings it in three way: oligohydramnios, normal, polyhydramnios. Then, deepest vertical pocket (DVP) evaluation was performed, consisting in measuring vertically the largest pocket free of fetal structures or cord with the ultrasound probe positioned parallel to sagittal plane^2,3^. This measurement was expressed in centimeters. Finally, the sonographer performed the amniotic fluid index (AFI) evaluation which is the sum (in centimeters) of the four largest vertical pockets of amniotic fluid, measured in each of the 4 uterus quadrants defined relative to the umbilicus and free of fetal structures or cord^4,5^.

Non-inclusion criteria were multiple pregnancies, maternal chronic pathology and fetal malformation. Small for gestational age or macrosomic fetuses were not excluded and participated in the study. All these prospectively collected measurements constituted our primary database. According to French law in the context of observational studies, obtaining the written consent of the patient was not necessary. Every patient had been informed of the study by the sonographer and by a newsletter. The patient’s data may be included in the study if they did not object to their anonymous use. This study received approval by the French ethics committee under the notification number 2016/71.

### Statistical analysis

Quantitative variables were described in the study population with means and standard deviations (SD) or median and interquartiles (Q25-Q75) depending on the distribution tested with the Shapiro-Wilk statistic. For categorical variables, the comparisons of percentage were made with chi-square test or Fisher’s exact test if chi-square was not valid. Normality distribution of variables was assessed using Shapiro-Wilk test and Q-Q plot method (supplementary materials). According to the normality of the distributions, comparisons of means between groups were performed using Student test or Mann Whitney rank sum test.

To evaluate the relationship between DVP or AFI and different maternal-fetal parameters, univariate and multivariate analysis using linear regression models were performed. Studied parameters were maternal age, BMI, fetal sex, tobacco use, nulliparity, nulligravidity and EFW. For the dependant variable SM, a logistic regression was performed and the Odds-ratio (OR) with their 95% confidence intervals (95% CI) were reported. The variables included in the logistic or linear models were variables, which have been shown previously to be associated with studied dependent variable (SM, DVP or AFI). The α-to-enter in the model was set at 0.20. The EFW, the maternal age and the BMI before pregnancy were included in all models. The EFW was modelled either in percentile in three categories (<10^th^ percentile or small for gestational age (SGA); 10–90^th^ percentile or normally grown foetuses and; >90^th^ or large for gestational age (LGA)), or in Z-score according to gestational age^28,29^. BMI was stratified in 5 groups in the models (<18.5 kg/m^2^, 18.5–24.9 kg/m^2^ (reference), 25–299 kg/m^2^, 30–34.9 kg/m^2^ and, ≥35 kg/m^2^. All analyses were two-tailed, with a p value of <0.05 considered statistically significant.

Statistical analysis was performed using SAS® Enterprise Guide software (version 7.12)^30^ and graphs were generated using R statistical software^31^ (www.r-project.org, version 3.4.3) with ggplot2 package^32^ (version 2.2.1).

## Results

**Figure 1:**
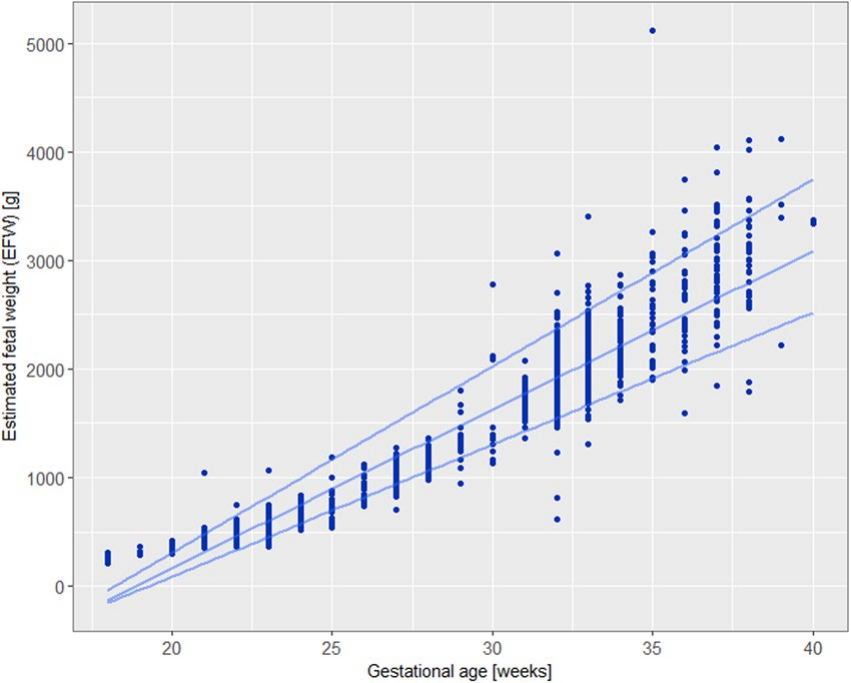
Distribution of Estimated Fetal Weight (grams) according to gestational age (weeks). 5^th^, Median and 95^th^ smooth percentiles are presented with the 3 lines.

During the two-week period, 1667 ultrasound scans were performed by 65 operators and included in the study. Mean maternal age was 31.6 years+/− 5.0 (range 18–46 years) and mean BMI at the beginning of pregnancy was 25.3 kg/m^2^+/− 4.9 (range 16.1–55.2). Distribution of BMI was as followed: <18.5 kg/m^2^ (2.5%), 18.5–24.9 kg/m^2^ (54%), 25–29.9 kg/m^2^ (28.4%), 30–35 kg/m^2^ (10.5%), >35 kg/m^2^ (4.6%). Thirty-eight percent of women were experiencing their first pregnancy, 47% were nulliparous, and 10.4% continued to smoke during their pregnancy. Ultrasound scan were performed at every gestational age from 18 to 40 weeks of gestation but more often at key gestational age corresponding to usual timing of French routine ultrasound: 1.5% under 21 weeks, 35% from 21–23 weeks, 13% from 24–30 weeks, 38.5% from 31–33 weeks, 7% from 34–36 weeks and 5% at term (37–40 weeks). Fetuses were female in 50%, male in 45% and undetermined or non-reported in 5%. EFW mean was 1,397.9 g +/−840.5 (range 209–5,119). The graph showing the distribution of EFW (median, 5^th^ and 95^th^ percentile) according to gestational age (in weeks) is presented in Fig. 1.

Due to the bimodal distribution of our data, grouped around second and third trimester of pregnancy corresponding to usual timing of French routine ultrasound, were decided to split our data in two. Second trimester corresponded to data ranging from 18 to 28 weeks and 6 days (n = 787); Third trimester corresponding to data from 29–40 weeks (n = 880).

**Figure 2:**
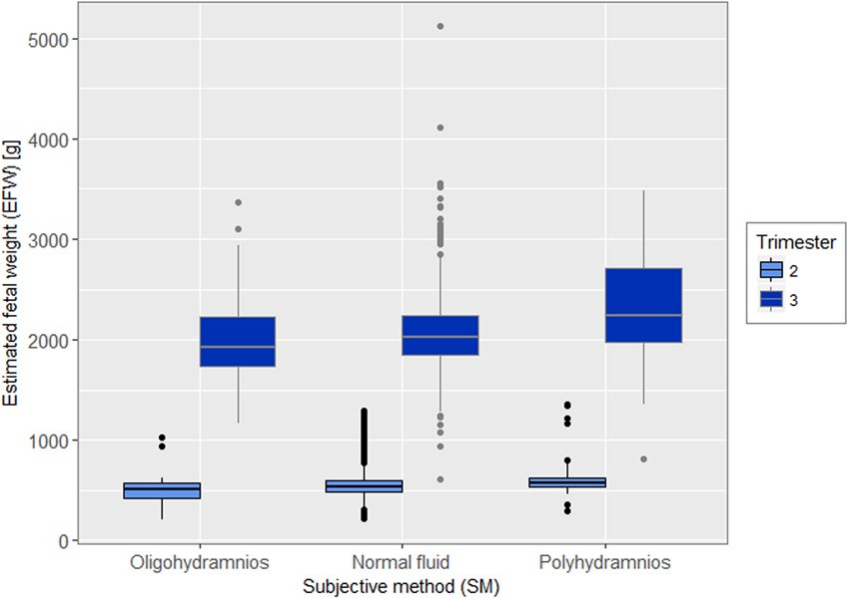
Distribution of Estimated Fetal Weight (presented in box plot) stratified by 2nd (18–28 weeks) and 3rd (29–40 weeks) trimester of pregnancy in each category of amniotic fluid according to Subjective Method (SM) evaluation.

Global amniotic fluid subjective evaluation (SM) classification was as followed: oligohydramnios (5.3%), normal fluid (86.8%) and polyhydramnios (7.9%) respectively. Distribution of oligohydramnios and polyhydramnios significantly differed from 2^nd^ versus 3^rd^ trimester, with 1.9% and 7% versus 9.2% and 9% respectively (p < 0.001). When analyzing SM categories according to EFW categories, oligohydramnios were more prevalent in the SGA category with 19.4% (versus 4.5% for normally grown fetuses and 1.4% for LGA) whereas polyhydramnios were more frequent in the LGA group with 10.8% (versus 5.4% for SGA and 7.9% for normally grown fetuses) (p < 0.001). Univariate analysis of the association between SM and EFW differed according to 2^nd^ or 3^rd^ trimester and according to EFW modeling. When EFW was modeled in z-score, SM classes was significantly associated with EFW, both in 2^nd^ and 3^rd^ trimester with p = 0.005 and p < 0.0001 respectively. When EFW was modeled in percentile (3 categories), SM classes was not significantly associated with EFW in the 2^nd^ trimester (p = 0.42), but remained significant in the 3^rd^ trimester (p < 0.0001). The distributions of EFW according to 2^nd^ and 3^rd^ trimester in each SM category are presented in Fig. 2.

**Figure 3:**
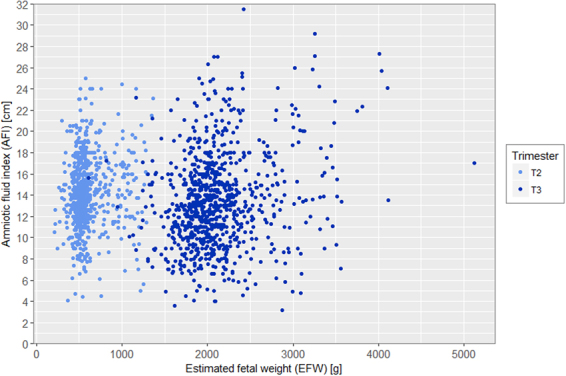
Distribution of deepest vertical pocket (DVP) according to estimated fetal weight (EFW) and stratified by 2nd (18–28 weeks) and 3rd (29–40 weeks) trimester of pregnancy.

**Figure 4:**
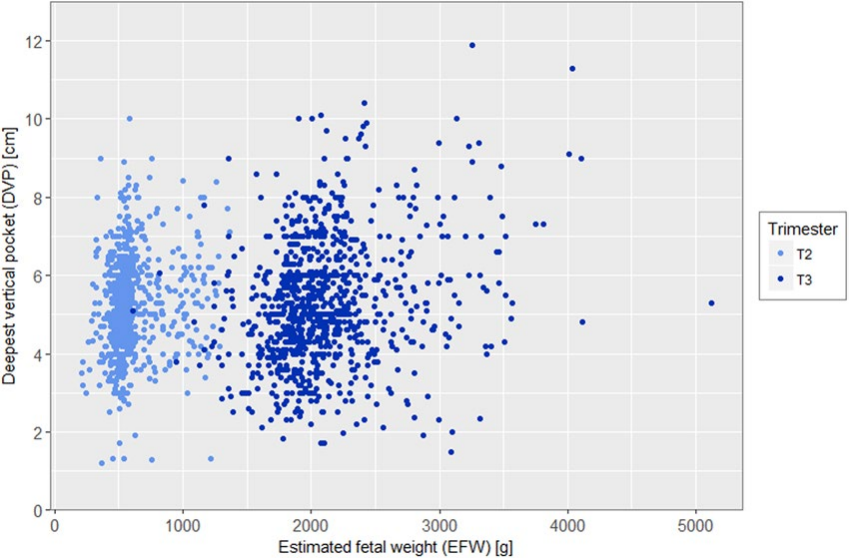
Distribution of amniotic fluid index (AFI) according to estimated fetal weight (EFW) and stratified by 2nd (18–28 weeks) and 3rd (29–40 weeks) trimester of pregnancy.

**Figure 5:**
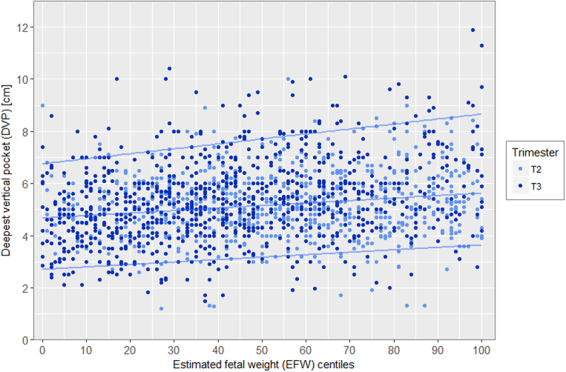
Distribution of deepest vertical pocket (DVP) according to estimated fetal weight (EFW) in centiles and stratified by 2nd (18–28 weeks) and 3rd (29–40 weeks) trimester of pregnancy.

**Figure 6:**
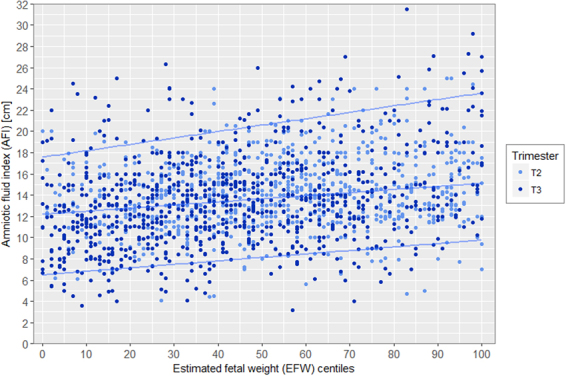
Distribution of amniotic fluid index (AFI) according to estimated fetal weight (EFW) in centiles and stratified by 2nd (18–28 weeks) and 3rd (29–40 weeks) trimester of pregnancy.

Distribution of deepest vertical pocket (DVP) and amniotic fluid index (AFI) according to EFW and stratified by trimester are presented in Figs 3 and 4 showing the bimodal distribution. In the Figs 5 and 6, distribution of DVP and AFI was also presented according to EFW in centiles in order to control for gestation (with lines represeting median, 5^th^ and 95^th^ percentile). Presenting the density of DVP and AFI (supplementary materials) in histograms, stratified by trimester, showed a Gaussian appearance of the data, without statistical confirmation of the normality of the distribution (Shapiro Wilk test; p < 0.0001 and p < 0.0001 respectively). However, due to the Gaussian appearance of the distribution, to the high number of observations and finally to the Q-Q plot analysis (supplementary materials) the hypothesis of normality of the data has been preserved. Univariate analysis of the association between DVP and EFW differed according to 2^nd^ or 3^rd^ trimester and according to EFW modeling. When EFW was modeled in z-score, DVP was significantly associated with EFW, both in 2^nd^ and 3^rd^ trimester with p = 0.02 and p < 0.0001 respectively. When EFW was modeled in percentile (3 categories), DVP was not significantly associated in the 2^nd^ trimester (p = 0.07), but remained significant in the 3^rd^ trimester (p < 0.001). As for SM and DVP, univariate analysis of the association between AFI and EFW differed according to 2^nd^ or 3^rd^ trimester and according to EFW modeling. When EFW was modeled in z-score, AFI was significantly associated with EFW, both in 2^nd^ and 3^rd^ trimester with p = 0.002 and p < 0.0001 respectively. When EFW was modeled in percentile (3 categories), AFI was not significantly associated with EFW in the 2^nd^ trimester (p = 0.06), but remained significant in the 3^rd^ trimester (p < 0.001).

Detailed results of univariate and multivariate analysis are presented in Tables 1 to 4.

**Table 1:** Univariate and multivariate logistic regression analysis of the subjective methods (SM) in the second trimester (T2).

	T2							
	Univariate Analysis (reference = Normal fluid)				Multivariate Analysis (reference = Normal fluid)			
	Oligohydramnios		Polyhydramnios		Oligohydramnios		Polyhydramnios	
	OR [95% CI]	*p*	OR [95% CI]	*p*	OR [95% CI]	*p*	OR [95% CI]	p
**Age (years)**	0.995 [0.895–1.106]	**0.927**	1.037 [0.978–1.100]	**0.219**	0.996 [0.894–1.110]	**0.944**	1.016 [0.952–1.085]	**0.636**
**BMI classes**		**0.913**		**0.091**		**0.902**		**0.153**
<18.5	<0.001 [<0.001–>999.9]	0.986	<0.001 [<0.001–>999.9]	0.983	<0.001 [<0.001–>999.9]	0.986	<0.001 [<0.001–>999.9]	0.984
18.5–24.9	—	—	—	—	—	—	—	—
25–29.9	1.662 [0.520–5.314]	0.391	0.803 [0.382–1.685]	0.561	1.692 [0.528–5.417]	0.376	0.773 [0.351–1.705]	0.524
30–35	1.016 [0.123–8.417]	0.988	1.226 [0.455–3.303]	0.687	1.150 [0.138–9.601]	0.897	1.223 [0.442–3.388]	0.698
>35	1.959 [0.233–16.488]	0.536	3.310 [1.332–8.226]	0.01	2.137 [0.249–18.370]	0.489	3.340 [1.209–9.228]	0.02
**Fetal sex**		**0.878**		**0.064**		—		**0.091**
Female	—	—	—	—	—	—	—	—
Male	1.103 [0.316–3.846]	0.878	1.776 [0.967–3.264]	0.064	—	—	1.731 [0.916–3.273]	0.091
**Smoker**		**0.653**		**0.857**		—		—
No	—	—	—	—	—	—	—	—
Yes	0.626 [0.081–4.854]	0.653	1.085 [0.447–2.631]	0.857	—	—	—	—
**Nulliparity**		**0.822**		**0.086**		—		**0.095**
No	—	—	—	—	—	—	—	—
Yes	0.885 [0.304–2.578]	0.822	0.590 [0.323–1.077]	0.086	—	—	0.555 [0.278–1.107]	0.095
**Nulligravidity**		**0.892**		**0.756**		—		—
No	—	—	—	—	—	—	—	—
Yes	0.927 [0.307–2.796]	0.892	0.910 [0.502–1.650]	0.756	—	—	—	—
**EFW percentiles**		**0.882**		**0.337**		**0.883**		**0.626**
<10	1.695 [0.214–13.436]	0.617	0.501 [0.067–3.773]	0.502	1.697 [0.211 – 13.633]	0.619	0.449 [0.056–3.572]	0.449
10–90	—	—	—	—	—	—	—	—
>90	<0.001 [<0.001–>999.9]	0.977	1.803 [0.729–4.458]	0.202	<0.001 [<0.001–>999.9]	0.976	1.339 [0.476 – 3.768]	0.58

**Table 2:** Univariate and multivariate logistic regression analysis of the subjective methods (SM) in the second trimester (T3).

	T3							
	Univariate Analysis (reference = Normal fluid)				Multivariate Analysis (reference = Normal fluid)			
	Oligohydramnios		Polyhydramnios		Oligohydramnios		Polyhydramnios	
	OR [95% CI]	*p*	OR [95% CI]	*p*	OR [95% CI]	*p*	OR [95% CI]	p
**Age (years)**	0.961 [0.910–1.014]	**0.148**	1.038 [0.984–1.096]	**0.17**	0.953 [0.900–1.008]	**0.093**	1.042 [0.986 – 1.101]	**0.14**
**BMI classes**		**0.477**		**0.253**		**0.353**		**0.184**
<18.5	<0.001 [<0.001–>999.9]	0.990	<0.001 [<0.001–>999.9]	0.990	<0.001 [<0.001–>999.9]	0.990	<0.001 [<0.001–>999.9]	0.990
18.5–24.9	—	—	—	—	—	—	—	—
25—29.9	0.976 [0.535–1.783]	0.938	1.744 [0.923–3.294]	0.087	0.981 [0.523–1.837]	0.951	1.840 [0.962–3.518]	0.065
30–35	0.570 [0.212–1.534]	0.266	2.224 [1.041–4.754]	0.039	0.630 [0.230–1.724]	0.368	2.473 [1.139 – 5.371]	0.022
>35	1.901 [0.721–5.017]	0.194	0.951 [0.209–4.322]	0.948	2.370 [0.869–6.465]	0.092	1.092 [0.237 – 5.034]	0.91
**Fetal sex**		**0.924**		**0.635**		—		—
Female	—	—	—	—	—	—	—	—
Male	0.973 [0.555–1.706]	0.924	0.873 [0.500–1.527]	0.635	—	—	—	—
**Smoker**		**0.988**		**0.250**		—		—
No	—	—	—	—	—	—	—	—
Yes	0.994 [0.433–2.281]	0.988	1.535 [0.741–3.183]	0.250	—	—	—	—
**Nulliparity**		**0.729**		**0.529**		—		—
No	—	—	—	—	—	—	—	—
Yes	1.10 [0.644–1.877]	0.729	0.861 [0.499–1.488]	0.529	—	—	—	—
**Nulligravidity**		**0.308**		**0.865**		—		—
No	—	—	—	—	—	—	—	—
Yes	1.326 [0.770–2.282]	0.308	0.952 [0.540–1.679]	0.865	—	—	—	—
**EFW percentiles**		**<0.0001**		**0.936**		**<0.0001**		**0.862**
<10	4.462 [2.343–8.500]	<0.0001	0.832 [0.287–2.409]	0.734	4.736 [2.401–9.342]	<0.0001	0.822 [0.279 – 2.423]	0.722
10–90	—	—	—	—	—	—	—	—
>90	0.564 [0.074–4.309]	0.581	0.894 [0.203–3.933]	0.882	0.620 [0.079–4.861]	0.649	0.717 [0.159 – 3.226]	0.664

**Table 3:** Univariate and multivariate linear regression analysis for the deepest vertical pocket method (DVP) stratified by trimester.

	T2						T3					
	Univariate Analysis			Multivariate Analysis			Univariate Analysis			Multivariate Analysis		
	Coefficient	SE	*p*	Coefficient	SE	*p*	Coefficient	SE		Coefficient	SE	*p*
Age (years)	0.026	0.008	**0.078**	0.018	0.009	**0.050**	0.013	0.011	**0.013**	0.021	0.011	**0.059**
BMI classes			**0.240**			**0.142**			**0.012**			**0.026**
<18.5	0.035	0.281	0.899	0.108	0.290	0.708	−0.377	0.351	0.282	−0.239	0.344	0.487
18.5–24.9	—	—	—	—	—	—	—	—	—	—	—	—
25–29.9	−0.103	0.105	0.325	−0.165	0.109	0.131	0.309	0.122	0.011	0.310	0.120	0.010
30–35	−0.164	0.166	0.325	−0.211	0.171	0.216	0.442	0.168	0.009	0.417	0.166	0.012
>35	0.364	0.21	0.084	0.334	0.217	0.125	0.231	0.262	0.378	0.152	0.261	0.561
Fetal sex			**0.029**			**0.023**			**0.343**			-
Female	—	—	—	—	—	—	—	—	—	—	—	—
Male	0.197	0.090	0.029	0.212	0.092	0.023	0.103	0.109	0.343	—	—	—
Smoker			**0.756**			**—**			**0.695**			—
No	—	—	—	—	—	—	—	—	—	—	—	—
Yes	0.044	0.144	0.756	—	—	—	0.068	0.173	0.695	—	—	—
Nulliparity			**0.914**			**—**			**0.026**			**0.597**
No	—	—	—	—	—	—	—	—	—	—	—	—
Yes	−0.009	0.088	0.914	—	—	—	−0.237	0.106	0.026	−0.095	0.18	0.597
Nulligravidity			**0.729**			**—**			**0.035**			**0.988**
No	—	—	—	—	—	—	—	—	—	—	—	—
Yes	−0.031	0.090	0.729	—	—	—	−0.231	0.109	0.035	−0.002	0.185	0.988
EFW percentiles			**0.067**			**0.130**			**<0.0001**			**<0.0001**
<10	−0.222	0.228	0.329	−0.268	0.238	0.261	−0.808	0.177	<0.0001	−0.792	0.181	0.0001
10–90	—	—	—	—	—	—	—	—	—	—	—	—
>90	0.321	0.159	0.040	0.233	0.166	0.160	1.042	0.247	<0.0001	0.964	0.248	<0.0001

**Table 4:** Univariate and multivariate linear regression analysis for the amniotic fluid index method (AFI) stratified by trimester.

	T2						T3					
	Univariate Analysis			Multivariate Analysis			Univariate Analysis			Multivariate Analysis		
	Coefficient	SE	*p*	Coefficient	SE	*p*	Coefficient	SE	*p*	Coefficient	SE	*p*
**Age (years)**	**0.016**	0.024	0.490	0.013	0.026	**0.629**	0.056	0.03	**0.06**	0.039	0.03	**0.191**
**BMI classes**			**0.087**			**0.082**			**0.392**			0.320
<18.5	−0.22	0.757	0.772	−0.308	0.786	0.695	−0.114	0.978	0.907	0.129	0.948	0.891
18.5–24.9	—	—	—	—	—	—	—	—	—	—	—	—
25—29.9	−0.124	0.283	0.661	−0.338	0.296	0.255	0.33	0.341	0.335	0.322	0.34	0.344
30–35	−0.567	0.448	0.206	−0.714	0.462	0.122	0.889	0.469	0.907	0.941	0.468	0.045
>35	1.332	0.566	0.019	1.148	0.589	0.051	−0.091	0.732	0.900	−0.265	0.719	0.712
**Fetal sex**			**0.006**			**0.006**			0.090			**0.538**
Female	—	—	—	—	—	—	—	—	—	—	—	—
Male	0.670	0.243	0.006	0.687	0.251	0.006	0.509	0.300	0.090	0.185	0.300	0.538
**Smoker**			**0.104**			**0.185**			0.940			—
No	—	—	—	—	—	—	—	—	—	—	—	—
Yes	0.632	0.388	0.104	0.543	0.409	0.185	0.036	0.481	0.940	—	—	—
**Nulliparity**			**0.389**			—			0.210			—
No	—	—	—	—	—	—	—	—	—	—	—	—
Yes	−0.204	0.237	0.389	—	—	—	−0.370	0.294	0.210	—	—	—
**Nulligravidity**			**0.156**			**0.323**			**0.221**			—
No	—	—	—	—	—	—	—	—	—	—	—	—
Yes	−0.346	0.243	0.156	−0.268	0.271	0.323	−0.372	0.304	0.221	—	—	—
**EFWpercentiles**			**0.058**			**0.299**			**<0.0001**			**<0.0001**
<10	−0.771	0.616	0.211	−0.659	0.644	0.307	−2.10	0.485	<0.0001	−2.135	0.500	<0.0001
10–90	—	—	—	—	—	—	—	—	—	—	—	—
>90	0.825	0.423	0.051	0.493	0.448	0.271	4.320	0.677	<0.0001	4.058	0.690	<0.0001

For SM evaluation, when using EFW percentile categories, no variable tested was associated with an oligohydramnios or a polyhydramnios in the 2^nd^ trimester (Table 1). Some variables (such as BMI < 18.5, or EFW > 90^th^ percentile in case of oligohydramnios) were so scarce that logistic regression modeled was not able to compute precise value for odds ratio (OR). In the 3^rd^ trimester, oligohydramnios found by SM method was significantly associated with SGA fetuses (p < 0.0001) whereas polyhydramnios found by SM was not associated with any variable including EFW in percentile (Table 2). When switching EFW modeling into Z-score in the logistic model, oligohydramnios found by SM at 2^nd^ or 3^rd^ trimester was significantly associated with decreasing Z-score (p < 0.03 and p < 0.001); whereas polyhydramnios found by SM remained significant only in the 2^nd^ trimester with increasing Z-score (p = 0.047).

An increase in DVP was found to be, in the 2^nd^ trimester, associated with an increase in maternal age (p = 0.05) and fetal male sex (p = 0.023) but not with EFW in percentile (p = 0.13); whereas in the 3^rd^ trimester, increasing DVP was associated strongly with EFW in percentile (more LGA and less SGA) (p < 0.0001) and BMI (overweight women and class 1 obesity) (p = 0.03). When using Z-scores of EFW, increasing DVP remained significantly associated with increasing Z-score of EFW regardless of the trimester (p = 0.04 and p < 0.001 respectively).

Finally, an increase in AFI in the 2^nd^ trimester, was associated only with fetal male sex (p = 0.006), but not with EFW in percentile (neither SGA nor LGA) (p = 0.3); whereas in the 3^rd^ trimester, EFW in percentile alone (more LGA and less SGA) was strongly associated with an increase in AFI (p < 0.0001). With the use of Z-scores of EFW, as for DVP, increasing AFI remained significantly associated with increasing Z-score of EFW regardless of the trimester (p = 0.02 and p < 0.001 respectively).

## Discussion

### Main Findings

Based on large population of patients scanned during a short period of time and at various gestational ages, this study showed that all three methods of amniotic fluid evaluation are significantly associated to estimated fetal weight. Other factors such as fetal male gender or maternal BMI (overweight and class I obesity) may also have an impact on the amount of amniotic fluid. SM was the method that was least often associated with estimated fetal weight especially in case of polyhydramnios showing its strong dependence to operator’s experience. DVP and AFI appeared equivalent except that maternal-fetal factors seemed to have a higher impact in DVP than AFI.

### Interpretation

To evaluate the association between amniotic fluid measurement and fetal biometry, we asked the sonographer to perform all three existing methods. We found a significant association between every method and EFW but with a different strength according to trimester stratification and EFW modeling methods. We know, from a statistical point of view that, according to the modeling method used for a continuous parameter, the results in term of significance could vary. Using Z-score of EFW in the regression model gives the advantage of having a boundless normally distributed variable with a lot of information, but less easy to interpret from a clinical point of view. On the opposite, using EFW percentile categories (SGA, normal, LGA) creates a loss of statistical information but increases the easiness of clinical interpretation. Therefore, for the second trimester analysis, where the number of SGA and LGA is small compared to third trimester, using EFW in percentile gives less significant results than Z-scores.

In France, AFI still remain a popular method and largely performed even though it has been demonstrated that DVP evaluation was more reliable^10,13,14^. As quoted by Magann *et al*.^10,13,14^, the use of AFI would result in an increase in the false positive rate for a diagnosis of oligohydramnios, in comparison with the single deepest vertical pocket method. In contrast, Kofinas *et al*. found that AFI was correlated to EFW percentile in the third trimester of pregnancy regardless of mother diabetic status^33^. Although no explanation has been given for pregnancies in non-diabetic patients, it has been postulated that pregnant fetuses of diabetic patients spend more time breathing than swallowing; and it is not possible to breathe and swallow at the same time. Then, the authors postulated that fetuses of diabetic patients swallow less fluid than normal. Thus, in diabetic pregnancies, it may be necessary to consider the EFW of the fetus when interpreting variations of amniotic fluid across gestational ages. However, this study does not indicate whether this correlation is strong enough to be considered and applied clinically. In our study, the goal was not compare directly the performance of one method to another. This has been previously performed and DVP seems to be more reliable, more representative of real amniotic fluid volume and the only method associated with a reduction rate of unnecessary induction of labor and of cesarean delivery for fetal distress^34^. In our study, based in a population free from gestational diabetes, DVP and AFI were both influenced by EFW. We confirmed Kofinas’ findings and we also found the same association with DVP in 3^rd^ trimester.

The least often association for SM with EFW could rely on the fact that only trained sonographer, are able to appropriately use this method. Besides, SM evaluation is not designed to accurately discriminate small variation of fetal weight and therefore is not appropriate when searching for that kind of association. In that situation, it fails to be strongly associated to parameters, due to the structure of this variable (categorical). It is more used to rapidly interpret the amount of amniotic fluid and needs to be confirmed by a precise measurement in case of suspected oligohydramnios or polyhydramnios.

Results of significant association between DVP/AFI and EFW confirmed our preliminary hypotheses. In the absence of maternal metabolic disease, fetuses with increased EFW would have had an increased DVP or AFI value. However, this association even statistically significant is not unique and other factors (male fetus, BMI, maternal age) also have an impact in the variation of the amount of amniotic fluid. This may be explained by the fact that fetal swallowing and fluid reabsorption by the mother are capable of maintaining an approximately stable amount of fluid irrespective of the amount of fetal urine produced. It can also be assumed that fetal urine production is finally only slightly influenced by the weight of the fetus. Previous studies by Adeyekun *et al*.^9^, in 2013 with 253 patients, or by Owen *et al*.^11^ in 2002 with 274 patients, found no correlation between EFW and AFI, either in the overall population or in the gestational age-separated subgroups, which is discordant to our results. However, these studies did not investigate association by using DVP, and we can assume that the number of patients was not sufficient to demonstrate a significant association. Despite the recognized limitations of the ultrasound measurement of the AFI and DVP, these measures remain an important component of antenatal fetal assessment when combined with other biophysical parameters. Therefore, it seems wise to continue to consider the variation of the AFI or DVP rather than SM alone.

Some adjustment on specific variable retrieved interesting results. Tobacco smoking, nulliparity and nulligravidity, even associated with a p value less than 0.2 for some univariate analysis, ever reached significance in the multivariate analysis. Compared to national French perinatal data^35^, the patients in our sample were older (31.6 years versus 30.3 years), had a higher BMI (overweight, class I and II/III obesity: 28.4%, 10.5%, 4.6% versus 20.2%, 8.1% and 3.7% respectively), smoked less tobacco (10.4% versus 16.6%) and were more often nulliparous (46.8% versus 42.2%). Therefore, our “low risk population” according to the study design might be slightly at higher risk than expected. However, we believe that non-significance of the aforementioned variables might be probably due to the strong impact of some variables such as EFW in the model.

A significant impact of BMI was observed for DVP assessed in the overweight and class I obesity group. In obese or overweight patients, literature has shown that the risk of macrosomia was strongly increased (OR 1.7–2.4) independently of the presence of gestational diabetes^36^. We could assume that increasing BMI is associated with increased EFW also associated with increased amniotic fluid volume. But, as an adjustment on EFW was performed, another possible assumption is that obese women do have a bigger placenta that produces higher amniotic fluid volume than smaller ones. The fact that this association was only observed with DVP and not AFI could be related to more difficult ultrasound examination in obese than in normal BMI patients^37^. The margins of error in the measures for the calculation of AFI may be greater, explaining why the association is not detected with that method.

Perni *et al*.^12^ found, in the third trimester of pregnancy, a significant correlation between EFW and AFI only in female fetuses (p < 0.001; r = 0.31). The correlation was significant for both fetal genders only after 38 weeks of gestation (p = 0.03 and r = 0.30). These results are not consistent with our results since we found an association between male fetus and DVP or AFI in the second trimester. Again, as for BMI, male fetuses are bigger than girl and therefore produce higher amount of amniotic fluid. But, as an adjustment on EFW was performed, we could then only assume that fetal urine production is probably higher in male fetus.

### Strengths and Limitations

Several strengths of our study should be mentioned. This is a large prospective multicentre study with experienced sonographers evaluating all the three methods of amniotic fluid evaluation when other studies mainly focused on AFI. Sonographers participating in the study had first to take an online training course reviewing the aims of the study, the inclusion criteria and the methodology to assess the amniotic fluid through the three methods, in order to reduce inter-observer variability. Only sonographers who completed the course and passed the final test were eligible to participate in the study. The large number of ultrasound scans performed in this study (1667 scans) enables us to produce precise and robust evaluation of associations. It gave us the power to observe relationship between parameters that were in other studies not visible due to the lack of power. Moreover, we studied the association over a long period of pregnancy ranging from 18 to 40 weeks to cover the maximum pregnancy time, unlike the other studies that were mainly limited to the third trimester of pregnancy. Several papers have examined the volume of amniotic fluid by gestational age, but with ultrasound data coming from a few hundred patients. Magann *et al*.^14^, for example, published percentile curves for DVP and AFI as a function of gestational age with data from 291 patients. Because of the large number of patients included in our study, we believe that these measurements can become a good database and can be used, later, to create new AFI and DVP percentile curves according to gestational age. However, a selection of very low risk patients with an EFW ranging from 10 to 90^th^ percentile would be the first step of this study. Then, it would be interesting to compare these curves with other curves published in the literature.

Some weakness may also be mentioned. Quantitative assessment methods such as DVP and AFI have the theoretical advantage of being reproducible and comparative. However, the measures themselves and the choice of the tank are largely subjective and vary greatly from one observer to another. We still often find, for the same pregnancies at the same term, with great disparities in the values of AFI or DVP in particular according to the observers. Despite the precautions taken to prevent this inter-observer variability in the measurement of biometrics and the quantity of liquid, it will remain the main bias in our study because there were 65 different sonographers. This variability is inescapable but it shows the real life. Another limitation relies on the absence of flow chart. We do not have any information regarding the number and the characteristics of the patient that refused to be included in the study. We agree with the fact that it could have created a selection bias if many of potentially includable patients were not included. However, due to the high number of women included by a restricted number of sonographer during a very short period of time, we believe that the number of patient missed to be included is relatively low. Another limitation relies on the lack of information at birth for the fetus included. In fact, as the goal of this study was only to answer the question of a sonographic association at a precise point of time, we do not have the longitudinal follow up of those pregnancies and therefore we do not have any other information on birthweight. This could have created a classification bias if we consider that the informations provided by the sonographer were wrong. Finally we were not able to compare the three methods in order to find the best one; but this was not the aim of our study.

In conclusion, our study, based on a large amount of data obtained during a short period of time, demonstrates that every method for assessing amniotic fluid estimation was associated with estimated fetal weight with more accurate precision at the third trimester.

### Details of Ethics Approval

This study received approbation of French ethics committee under the notification number 2016/71.

## Electronic supplementary material

Supplementary information
