# Supplementary material for: Association between amniotic fluid evaluation and fetal biometry: a prospective French “Flash” study
Source: Sci Rep. 2018 May 4;8:7093. doi: 10.1038/s41598-018-25497-3 (PMC5935749; doi:10.1038/s41598-018-25497-3)
Supplement: Supplementary file 1 — Supplementary information [file 41598_2018_25497_MOESM1_ESM.doc]

**Supplementary information (supplementary Materials)**

Association between amniotic fluid evaluation and fetal biometry: a prospective French “Flash” study

Florent FUCHS*, MD,PhD^1,2^; Safa AOUINTI, PhD^3^; Manel SOUAIED, Midwife^4^; Valentin KELLER, MD^4^; Marie-Christine PICOT, MD, PhD^3^; Nicolas FRIES, MD^1,5^; Jean-Marc AYOUBI MD, PhD^4^, Olivier PICONE, MD,PhD4 (past affiliation),5,6,7 (new affiliation)

^1^Department of Obstetrics and Gynecology. Montpellier University Hospital Center, 371 Avenue du Doyen Gaston Giraud, Montpellier, France.

^2^Inserm, CESP Centre for research in Epidemiology and Population Health, U1018, Reproduction and child development, Villejuif,

^3^ Clinical Research and Epidemiology Unit (URCE), CHU Montpellier, Univ Montpellier, Montpellier, France.

^4^ Department of Obstetrics and Gynecology. Hopital Foch, 40 rue Worth, Suresnes, France.

^5^ Collège Français d'Echographie Foetale, CFEF, France,

^6^ EA2493, UFR des sciences de la santé Simone Veil, Université Versailles Saint Quentin en Yvelines

^7^ Department of Obstetrics and Gynecology. Louis Mourier Hospital, Paris Nord Val de seine University Hospitals, APHP, Paris-Diderot University, 178 rue des Renouillers Colombes, France.

# Corresponding Author*

Dr Florent FUCHS

Department of Obstetrics and Gynecology

CHU de Montpellier

Hopital Arnaud de Villeneuve

371, av. du Doyen Gaston Giraud

34295 MONTPELLIER cedex 5

Phone+33-6-65-84-94-80

Fax +33-4-67-33-64-68

Email: [f-fuchs@chu-montpellier.fr](mailto:f-fuchs@chu-montpellier.fr)


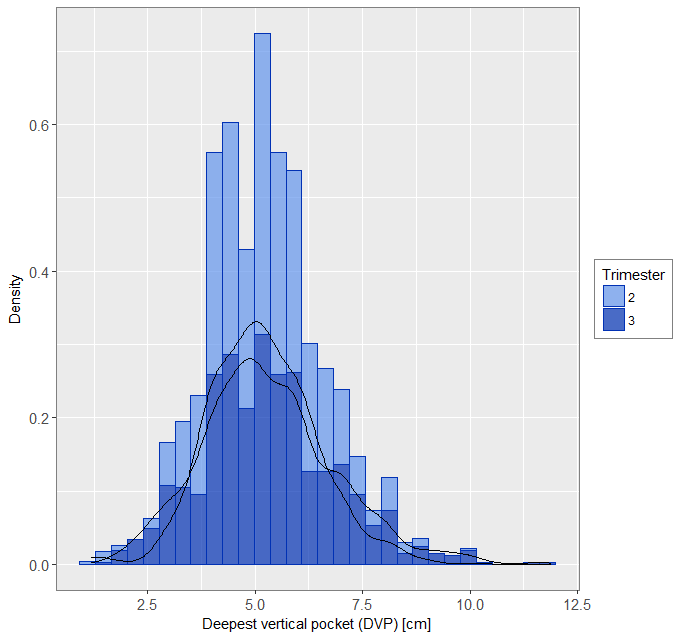


Histogram of the density of deepest vertical pocket (DVP) stratified by trimester


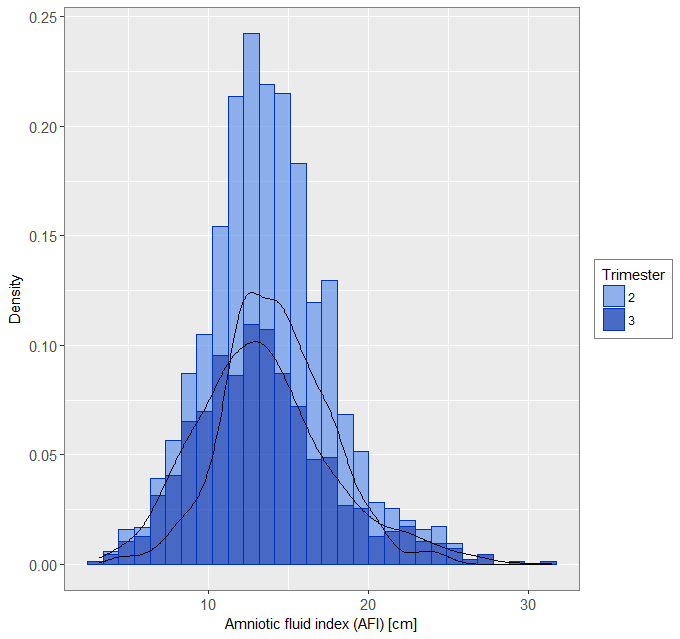


Histogram of the density of amniotic fluid index (AFI) stratified by trimester

**
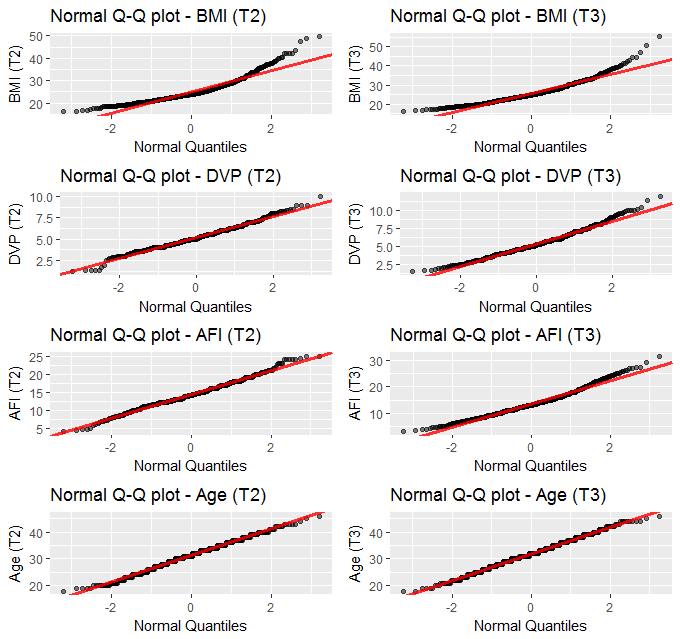
**

Q-Q plots presenting the distribution of BMI, DVP, AFI and maternal Age stratified by trimester and normalized by quantiles. Each distribution fitted the normal distribution quite well in each graph.
